# In reply to the letter to the editor regarding “Cemented versus uncemented hemiarthroplasty for femoral neck fractures in patients with neuromuscular diseases: a minimum of 2 years’ follow-up study”

**DOI:** 10.1186/s13018-021-02674-1

**Published:** 2021-08-25

**Authors:** Yuchuan Wang, Zhongzheng Wang, Siyu Tian, Zhanchao Tan, Yanbin Zhu, Wei Chen, Yingze Zhang

**Affiliations:** 1 Department of Orthopaedic Surgery, The 3rd Hospital of Hebei Medical University, Shijiazhuang, 050051 Hebei People’s Republic of China; 2 Orthopaedic Institution of Hebei Province, Shijiazhuang, 050051 Hebei People’s Republic of China; 3 Key Laboratory of Biomechanics of Hebei Province, Shijiazhuang, 050051 Hebei People’s Republic of China; 4 NHC Key Laboratory of Intelligent Orthopeadic Equipment, Shijiazhuang, 050051 Hebei People’s Republic of China; 5 Chinese Academy of Engineering, Beijing, 100088 People’s Republic of China

Dear Editor,

We appreciate Dr. Huang et al. for their thought-provoking and insightful comments about our recent publication [1], and we thank the Editor for providing us an opportunity to make a response. Our answers to the questions are as follows:

First, as with the quality of life, both the pain evaluation and the Harris hip score were obtained at the last follow-up. We explained it in the “Methods” section of the paper as follows: “The factors assessed were duration of surgery, intraoperative blood loss, perioperative and postoperative complications, pain, functional outcomes, quality of life at last follow-up, and survivorship.” I guess Dr. Huang et al. may have misunderstood our definition due to the inadequate expressions.

In our study, the intraoperative blood loss was assessed by anesthetists and recorded in the anesthesia record sheet. The intraoperative blood loss was measured as the sum of the blood in suction bottles (after subtracting the lavage fluid used during the surgery) and the total volume of blood lost in gauzes and surgical towels during the procedure. The electronic medical record includes the anesthesia record sheet from which the intraoperative bleeding was collected. We also described in the Methods section of the paper that data on procedure details were collected from the electronic medical record by a trained research associate who was blinded to the treatment allocation.

Dr. Huang et al. concluded that periprosthetic fractures are generally related to either inexperienced surgeons or inadequate surgical techniques. However, there is a dearth of literature that has fully studied whether periprosthetic fractures after hemiarthroplasty are related to inexperienced surgeons or inadequate surgical techniques. Although the surgeries were not performed by the same surgeon in our study, all operations were performed by experienced surgeons or residents supervised by an experienced surgeon. In a study on periprosthetic fractures after total hip arthroplasty requiring greater surgical technique, Solgaard et al. showed that there was no significant difference in periprosthetic fractures between experienced surgeons and trainees assisted by experienced surgeons, and that the surgeon’s experience had no significant influence on the periprosthetic fractures [2].

Second, one of the great concerns with bone cement is the occurrence of bone cement implantation syndrome (BCIS). Severe BCIS has a huge impact on early and late mortality, but BCIS is rare. The guideline has recently been issued on how to minimize the incidence of severe bone cement reactions [3]. In addition, several randomized trials have reported a trend toward lower 1-year mortality in the cemented group [4]. In our study, intraoperative death was not detected, which may be due to the limitation of the small sample size of the study. Although our results reflect similar mortality rates for uncemented hemiarthroplasty and cemented hemiarthroplasty, an uncemented implant may still be appropriate for those at very high risk of BCIS.

Finally, thanks again for your comments on this article, and we hope our reply will help answer the questions of Huang et al.

## Data Availability

Not applicable
